# COVID‐19 as a Potential Trigger for Tuberculosis: Insights From a Large‐Scale Japanese Insurance Database Analysis

**DOI:** 10.1002/jgf2.70139

**Published:** 2026-06-02

**Authors:** Daisuke Miyamori, Kotaro Ikeda, Sachi Nagasaka, Masanori Ito

**Affiliations:** ^1^ Department of General Internal Medicine Hiroshima University Hospital Hiroshima Japan

**Keywords:** COVID‐19, National Insurance Database, propensity score matching, tuberculosis

## Abstract

**Background:**

The long‐term impact of COVID‐19 on tuberculosis (TB) is concerning. This study assessed whether COVID‐19 was associated with an increased risk of active TB treatment risk in Japan using a nationwide propensity score–matched cohort design.

**Methods:**

A retrospective cohort study used Japan's National Insurance Database from January 2020 to December 2022. COVID‐19 patients were matched 1:1 with controls using propensity scores based on age, sex, immunosuppressants use, Charlson comorbidity index, related medical conditions, healthcare utilization and prior TB history. The primary outcome was initiation of isoniazid‐based therapy plus rifampin with a TB‐related ICD‐10 diagnosis code. Cox proportional hazards models estimated hazard ratios (HRs) for COVID‐19's effect on TB treatment initiation.

**Results:**

After matching, 3,097,422 individuals were included per group. During 8‐month median follow‐up, 886 participants initiated TB treatment. The HR among COVID‐19 infected individuals was 4.14 (95% CI: 3.51–4.89) versus matched controls. Subgroup analyses showed interaction between prior TB history and COVID‐19, with HR of 14.7 (5.35–40.2) in those with prior TB history, versus those without (HR 3.84, 95% CI 3.24–4.54). Risk remained elevated in participants without hospitalization (HR 3.96, 95% CI 3.35–4.68).

**Conclusions:**

This study provides robust population‐based evidence demonstrating a moderate association between prior COVID‐19 and subsequent initiation of active tuberculosis treatment, particularly in those with prior TB history.

## Graphical abstract

In a nationwide propensity score–matched cohort study using Japanese insurance claims data, prior COVID‐19 was associated with a fourfold higher risk of subsequent active tuberculosis treatment initiation. The association was particularly pronounced among individuals with prior tuberculosis history.

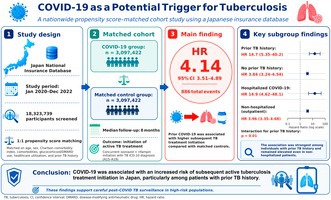

## 1 Introduction

COVID‐19, caused by SARS‐CoV‐2, has brought extensive challenges to global healthcare since its emergence in late 2019 [1, 2]. Beyond acute respiratory symptoms, the long‐term consequences of COVID‐19 on other infectious diseases have garnered growing attention [3, 4]. Tuberculosis (TB) is a major global health threat, causing more than one million deaths each year despite intense efforts at control [5, 6]. In Japan, although TB incidence has gradually declined over the past decades, it remains a significant public health concern, especially in the context of demographic shifts and rising comorbidities [7, 8].

Early observational data hinted that patients recovering from viral pneumonia might be more susceptible to secondary or reactivated infections, including TB [9]. This suspicion is rooted in immunological phenomena: SARS‐CoV‐2 disrupts multiple components of the immune system, from T‐lymphocyte depletion to hyperinflammatory states [10]. Moreover, COVID‐19's pathological impact on the lung environment, epithelial damage, microvascular changes, and dysregulated cytokine cascades, could potentially facilitate *Mycobacterium tuberculosis* reactivation or accelerated progression of latent TB [11, 12]. The interplay between chronic lung changes and immunosuppression is of special concern in older and comorbid populations, which comprise a large fraction of Japan's demographic profile [13].

Several recent studies have suggested a possible association between COVID‐19 and subsequent tuberculosis; however, large‐scale population‐based evidence using stringent outcome definitions remains limited. Concerns have also arisen that pandemic‐related disruptions in healthcare services and diagnostic pathways might delay detection of active TB cases [14, 15]. In Japan, the National Insurance Database captures large‐scale, claims‐based information on patient diagnoses, treatments, and procedures. Harnessing these data with rigorous matching methods offers a unique opportunity to estimate the comparative incidence of active TB between post‐COVID‐19 patients and non‐infected controls.

We designed this study with several objectives. First, we aimed to assess whether COVID‐19 infection is associated with elevated risk of active TB treatment initiation, using the combined start of isoniazid‐based therapy and rifampin with ICD‐10 codes to define active TB. Second, we investigated effect modification by demographic and medical status, such as glucocorticoid or DMARD use, and prior TB history [16, 17]. We applied a propensity score–matched (PSM) design to address confounding. Our findings can inform targeted TB surveillance and prophylactic measures in the post‐COVID population.

## 2 Methods

### 2.1 Study Design and Setting

We conducted a retrospective cohort study within Japan's National Insurance Database from January 2019 to December 2022. The study period was chosen to ensure adequate ascertainment of baseline covariates and prior tuberculosis history, as well as sufficient follow‐up time for the detection of incident active tuberculosis treatment after COVID‐19. In addition, this period was selected to minimize heterogeneity in healthcare‐seeking behavior and diagnostic practices, as changes in public health policy—particularly the reclassification of COVID‐19 to a lower‐risk category in Japan—may have influenced healthcare utilization patterns and diagnostic intensity. This database includes comprehensive claims data on outpatient and inpatient services, covering most residents through the universal insurance framework [18, 19]. Each individual has a unique identifier allowing longitudinal tracking of diagnoses and prescriptions, whereas personal data remain anonymized for privacy.

### 2.2 Participants

We identified individuals diagnosed with SARS‐CoV‐2 infection at any point between January 2020 and December 2022 using claims with COVID‐19‐specific reimbursement codes. In Japan, all confirmed cases are systematically reimbursed according to government‐funded pandemic measures, which facilitated consistent coding. Index month was defined as the month in which a qualifying COVID‐19 claim first appeared.

Potential controls were those with no recorded COVID‐19 claims at the same month. They were assigned a pseudo‐index month corresponding to the month of their matched COVID‐19 pair. Cases and controls had to be actively enrolled in the insurance system for ≥ 12 months prior to that index month for the assessment of comorbidities, medication usage, and healthcare utilization.

### 2.3 Propensity Score Matching

We used a 1:1 nearest‐neighbor PSM approach to match COVID‐19 patients to controls [20, 21]. Importantly, matching was performed within each index month so that temporal variations in infection dynamics, diagnostic practices, and health system utilization were minimized. Each control could only be used once.

We applied a logistic regression model to forecast COVID‐19 status using covariates that affect both tuberculosis risk and the probability of SARS‐CoV‐2 infection. Propensity scores were estimated using a logistic regression model, including age (5‐year categories), sex, prior tuberculosis history, Charlson Comorbidity Index (CCI), medical conditions which may risk for tuberculosis, immunosuppressive medication use (glucocorticoids and DMARDs), and healthcare utilization measured by annual medical visits before the index date (Table S1). Table S2 provides detailed ICD‐10 codes for comorbidities and the calculation of CCI, procedure codes for health care utilization, and medications for glucocorticoid and DMARDs categorization with definitions for each variable. Baseline medications were defined as more than four times prescriptions during the year before the index date to identify sustained rather than sporadic exposure and to capture clinically meaningful immunosuppressive status. After calculating propensity scores, we matched 1:1 with COVID‐19 case and the control having the nearest score (caliper 0.2 of logit, without replacement). Balance was evaluated using SMD, with a target of < 0.1 as an acceptable threshold.

### 2.4 Outcome

Initiation of co‐prescribed isoniazid plus rifampin and a concurrent ICD‐10 diagnosis code A15–A19 (tuberculosis) within the same billing cycle (or within 30 days) was used to define newly active TB treatment initiation (Table S3). This combined requirement minimizes the risk of including prophylactic regimens or historical codes without ongoing therapy, thereby indicating a more definitive treatment start for active TB. We excluded those who had initiated potential tuberculosis medications (Table S4) before the index month, ensuring that only new TB therapy episodes were counted.

As a secondary outcome, we evaluated each ICD‐10 code (A15–A19) with co‐prescribed isoniazid plus rifampin separately.

### 2.5 Follow‐Up and Censoring

Follow‐up began at the index month for each matched pair, and individuals were observed until one of three events: TB therapy initiation, reaching the end of the observation period in December 2022 (or 24 months post‐index), or loss to follow‐up (insurance discontinuation or death). Follow‐up was terminated if a participant contracted COVID‐19 after enrolment.

### 2.6 Statistical Analysis

All analyses were conducted using Stata (version 18.0, StataCorp, College Station, TX). Descriptive statistics summarized matched cohorts, and SMD were used to verify covariate balance. Kaplan–Meier curves and the log‐rank test evaluated unadjusted differences in time‐to‐TB therapy between COVID‐19 and control groups.

**A** Cox proportional hazards model then estimated hazard ratios (HRs) and 95% confidence intervals (CI) for the effect of COVID‐19 on TB treatment. We tested for effect modification across subgroups (demographic, medications and prior TB infection) by fitting interaction terms or using likelihood‐ratio (LR) tests [22]. To assess potential surveillance bias, we also descriptively compared follow‐up healthcare utilization between groups, including revisit frequency and chest imaging procedures after the index date.

### 2.7 Ethics Statement

This study was approved by the Ethical Committee of Hiroshima University (approval no.: E2020‐2024‐02). The Ministry of Health, Labour, and Welfare (MHLW) granted permission to access anonymized National Insurance Database records. Participant consent was waived due to retrospective de‐identification.

## 3 Results

### 3.1 Matching and Baseline

**FIGURE 1:**
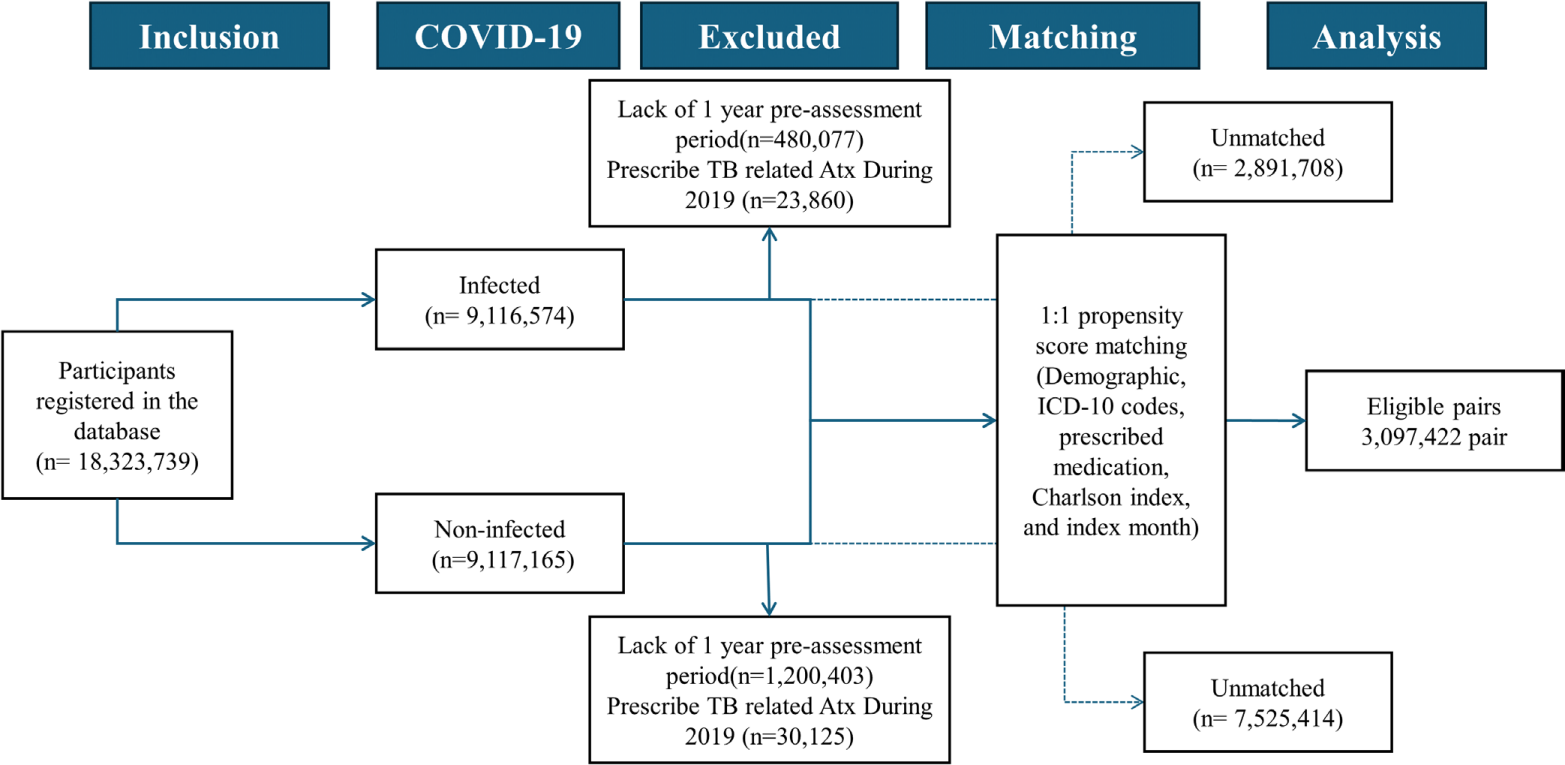
Flowchart of this study. Among 18,323,739 participants in this study, after 1:1 propensity score matching, 3,097,422 pairs were included in the main analysis.

From the initial pool, 6,194,844 individuals remained after 1:1 PSM. Cases of 3,097,422 for **C**OVID‐19 Group and **c**ontrol Group are matched (Figure 1). Table 1 summarizes the matched baseline characteristics. Most frequent age category in both groups was 70–74 years. Women represented 56% of the entire cohort. Glucocorticoid users were present in 1.5% of matched participants. DMARD users were found in 0.9%. Chronic Pulmonary disease appeared in 11%. Prior TB history was documented in 0.5% overall. After matching, SMD for all covariates was below 0.1, indicating acceptable balance.

**TABLE 1:** Baseline characteristics of the matched cohort.

Variable	Overall (*N* = 6,194,844)	Matched control (*N* = 3,097,422)	COVID‐19 Group (*N* = 3,097,422)	SMD
Hospitalized			61,587 (2.0%)	**NA**
Age category				0.00022
0–4 years	404,377 (7%)	202,183 (7%)	202,194 (7%)	
5–9 years	319,295 (5%)	159,643 (5%)	159,652 (5%)	
10–14 years	252,782 (4%)	126,417 (4%)	126,365 (4%)	
15–19 years	247,589 (4%)	123,790 (4%)	123,799 (4%)	
20–24 years	273,861 (4%)	136,925 (4%)	136,936 (4%)	
25–29 years	278,755 (4%)	139,365 (4%)	139,390 (5%)	
30–34 years	295,157 (5%)	147,522 (5%)	147,635 (5%)	
35–39 years	303,555 (5%)	151,738 (5%)	151,817 (5%)	
40–44 years	312,567 (5%)	156,249 (5%)	156,318 (5%)	
45–49 years	368,412 (6%)	184,189 (6%)	184,223 (6%)	
50–54 years	362,938 (6%)	181,480 (6%)	181,458 (6%)	
55–59 years	338,456 (5%)	169,193 (5%)	169,263 (5%)	
60–64 years	326,657 (5%)	163,336 (5%)	163,321 (5%)	
65–69 years	348,700 (6%)	174,402 (6%)	174,298 (6%)	
70–74 years	503,610 (8%)	251,899 (8%)	251,711 (8%)	
75–79 years	434,851 (7%)	217,524 (7%)	217,327 (7%)	
80–84 years	381,011 (6%)	190,521 (6%)	190,490 (6%)	
85+ years	442,271 (7%)	221,046 (7%)	221,225 (7%)	
Female	3,460,133 (55.9%)	1,730,101 (55.9%)	1,730,032 (55.9%)	0.00004
Charlson Index				−0.00050
0	2,364,211 (38%)	1,182,141 (38%)	1,182,070 (38%)	
1	1,270,860 (21%)	635,489 (21%)	635,371 (21%)	
2–3	1,272,204 (21%)	636,287 (21%)	635,917 (21%)	
4 or over	1,287,569 (21%)	643,505 (21%)	644,064 (21%)	
Comorbidity				
Diabetes	527,502 (8.5%)	263,431 (8.5%)	264,071 (8.5%)	−0.00074
AMI	149,602 (2.4%)	75,158 (2.4%)	74,444 (2.4%)	0.00150
CHF	1,070,205 (17.3%)	522,185 (16.9%)	548,020 (17.7%)	−0.0221
PVD	864,652 (14.0%)	441,295 (14.2%)	423,357 (13.7%)	0.0167
CEVD	1,016,682 (16.4%)	510,269 (16.5%)	506,413 (16.3%)	0.00336
Dementia	311,592 (5.0%)	138,888 (4.5%)	172,704 (5.6%)	−0.0499
CPD	699,579 (11.3%)	349,535 (11.3%)	350,044 (11.3%)	−0.00052
Rheumatoid	304,195 (4.9%)	156,017 (5.0%)	148,178 (4.8%)	0.0117
PUD	149,602 (2.4%)	75,158 (2.4%)	74,444 (2.4%)	−0.0158
HP/PAPL	96,307 (1.6%)	47,584 (1.5%)	48,723 (1.6%)	0.0247
RD	342,372 (5.5%)	170,849 (5.5%)	171,523 (5.5%)	−0.0265
LD	32,962 (0.5%)	16,033 (0.5%)	16,929 (0.5%)	−0.00398
AIDS	4423 (0.1%)	1955 (0.1%)	2468 (0.1%)	−0.0062
Cancer	813,127 (13.1%)	406,303 (13.1%)	406,824 (13.1%)	0.00060
Prior TB	30,590 (0.5%)	13,945 (0.5%)	16,645 (0.5%)	−0.0124
Medications				
GC	92,224 (1.5%)	45,579 (1.5%)	46,645 (1.5%)	−0.00284
DMARD	58,321 (0.9%)	28,528 (0.9%)	29,793 (1.0%)	−0.00423
Medical visits per year				0.00022
0–3	1,665,166 (27%)	832,511 (27%)	832,655 (27%)	
4–6	1,049,390 (17%)	524,503 (17%)	524,887 (17%)	
7–9	1,839,346 (30%)	919,876 (30%)	919,470 (30%)	
10 or over	1,640,942 (26%)	820,532 (26%)	820,410 (26%)	
Emergency	221,938 (3.6%)	110,575 (3.6%)	111,363 (3.6%)	−0.0047

Abbreviations: AIDS, acquired immune deficiency syndrome; AMI, acute myocardial infarction; CEVD, cerebrovascular disease; CHF, congestive heart failure; CPD, chronic pulmonary disease; DMARD, disease‐modifying antirheumatic drug; GC, glucocorticoid; HP/PAPL, hemiplegia or paraplegia; LD, liver disease; PUD, peptic ulcer disease; PVD, peripheral vascular disease; RD, renal disease; Rheumatoid, rheumatoid disease.

### 3.2 Main Analysis

**FIGURE 2:**
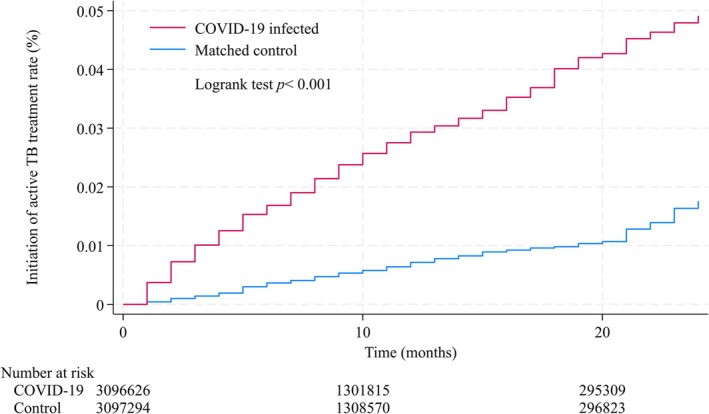
Kaplan–Meier analysis for composite outcome. Median follow‐up period was 8 months (interquartile range 4–12). Log‐rank test showed *p* < 0.001.

Figure 2 shows the Kaplan–Meier curve for the primary analysis. Table 2 shows the HRs for composite and secondary outcomes. During a median follow‐up of 8 months (interquartile range: 4 to 12 months), a total of 886 participants initiated the co‐prescribed isoniazid plus rifampin therapy, constituting the composite primary outcome. The HR for this outcome among COVID‐19 infected individuals was 4.13 (95% CI: 3.51–4.89) compared with matched controls, indicating a more than fourfold increased risk of active TB treatment initiation following COVID‐19.

**TABLE 2:** Primary and secondary outcome.

Outcome	Events	HR (95% CI)
Composite endpoint	886	4.14 (3.51–4.89)
**Secondary (ICD10‐code)**		
Respiratory tuberculosis, bacteriologically and histologically confirmed (A15)	62	4.18 (2.23–7.85)
Respiratory tuberculosis, not confirmed bacteriologically or histologically (A16)	609	3.56 (2.94–4.31)
Tuberculosis of nervous system (A17)	13	5.51 (1.22–24.9)
Tuberculosis of other organs (A18)	137	3.43 (2.30–5.12)
Miliary tuberculosis (A19)	23	22.1 (2.98–163.8)

*Note:* The composite primary outcome was defined as the co‐initiation of isoniazid‐based therapy plus rifampin, based on prescription data, in combination with the presence of any TB‐related ICD‐10 code (A15–A19). Secondary outcomes are based on specific ICD‐10 codes representing the site and diagnostic certainty of tuberculosis. Because patients could be assigned multiple TB‐related ICD‐10 codes simultaneously, the secondary outcome categories are not mutually exclusive. Therefore, the number of composite endpoint events is not expected to equal the arithmetic sum of the secondary outcome categories.

When evaluating individual ICD‐10 codes as secondary outcomes, a similarly elevated risk was observed. Specifically, the HR for bacteriologically or histologically confirmed respiratory TB (A15) was 4.18 (2.26–7.85), and for clinically diagnosed respiratory TB (A16) was 3.56 (2.94–4.31). The risk also appeared elevated for TB of other organs (A18) (HR: 3.42 [2.30–5.12]) and miliary TB (A19) (HR: 22.1 [2.13–163.8]), though estimates were less precise due to smaller event counts. Tuberculosis of the nervous system (A17) was rare, with only 13 events observed, yielding an HR of 5.52 (1.22–24.9).

### 3.3 Subgroup Analysis

Subgroup analyses are presented in Table 3. The association between COVID‐19 infection and subsequent initiation of tuberculosis (TB) treatment was observed consistently across all examined strata. In hospitalized patients, the HR shows 14.9 (95% CI: 4.61–48.1) compared with those matching control, whereas the HR shows 3.96 (95% CI: 3.35–4.68) among non‐hospitalized COVID‐19 patients.

**TABLE 3:** Subgroups analysis with likelihood‐ratio test.

Subgroup	Category	Number of participants	Events	HR (95% CI)	*p* for interaction
**Demographic**					
Sex	Male	2,734,711	517	4.09 (3.30–5.08)	0.87
	Female	3,460,133	369	4.21 (3.25–5.45)	
Age	0–19	1,224,043	12	4.94 (1.08–22.5)	0.14
	20–64	2,860,358	244	3.26 (2.42–4.38)	
	65 or over	2,110,443	630	4.66 (3.80–5.70)	
**Medications**					
GC	No	6,102,620	850	4.23 (3.57–5.02)	0.21
	Yes	92,224	36	2.61 (1.25–5.40)	
DMARD	No	6,136,523	870	4.14 (3.50–4.90)	0.98
	Yes	58,321	16	4.29 (1.22–15.06)	
Prior TB infection	No	61,34,434	814	3.84 (3.24–4.54)	0.01
	Yes	30,410	72	14.7 (5.35–40.2)	
Hospitalized		123,174	45	14.9 (4.62–48.1)	
Outpatient		6,071,670	841	3.96 (3.35–4.68)	

*Note:* Regarding hospitalization status, comparisons were made with each individual's matched pair. As the control group did not include hospitalized patients in this subgroup, the *p* value for interaction was not assessed.

Abbreviations: CI, confidence interval; DMARD, disease‐modifying antirheumatic drug; GC, glucocorticoid; HR, hazard ratio; TB, tuberculosis.

Among males, the hazard ratio (HR) was 4.09 (95% CI: 3.30–5.08), and among females, it was 4.21 (3.25–5.45), with no evidence of interaction by sex (p for interaction = 0.87).

There was also no significant interaction between the COVID‐19 infection and TB association and use of glucocorticoids (*p* = 0.21) or DMARDs (*p* = 0.98). HRs were 4.23 (3.57–5.02) for those not using steroids and 2.61 (1.26–5.40) for steroid users. Among those using DMARDs, the HR was 4.20 (1.20–14.7) compared with 4.14 (3.50–5.21) among non‐users.

A statistically significant interaction was found for history of tuberculosis infection (*p* for interaction = 0.01). Participants with a prior history of TB had a substantially higher HR of 14.7 (5.35–40.2), compared with 3.84 (3.24–4.54) in those without TB history, suggesting a heightened susceptibility to TB reactivation following COVID‐19 in this subgroup. The association of active TB treatment following COVID‐19 is broadly consistent across clinical subgroups, with the exception of those with a prior TB history.

To assess differences in diagnostic intensity after the index month, we descriptively compared follow‐up healthcare utilization between the COVID‐19 and matched control groups. As shown in Table S5, follow‐up visits, X‐ray examinations, and computed tomography examinations were more frequently recorded in the COVID‐19 group across all follow‐up periods.

## 4 Discussion

In this study, using a nationwide, propensity score–matched study involving approximately 3.1 million people in Japan, we demonstrated that a history of COVID‐19 was associated with the risk of initiating treatment for active tuberculosis by more than fourfold. The risk is particularly pronounced, rising by about 15 times, among individuals with a prior history of TB. This association of TB following COVID‐19 aligns with previous case reports and small series where TB developed within weeks to months after recovery [23, 24, 25] and with findings from systematic reviews noting reports of post‐COVID TB onset from various countries [25].

Most previous reports have been limited to case reports or small cohorts, and there have been few epidemiological data quantifying to what extent COVID‐19 is associated with the risk of TB reactivation or new onset. As a result, it has remained unclear whether COVID‐19 promotes the progression of TB [25]. This study helps bridge that gap by showing a strong association between prior COVID‐19 and initiation of TB treatment at the individual level, while partially adjusting for programmatic factors, such as healthcare access and diagnostic delays.

There is a significant interaction between prior TB history and COVID‐19. Among 886 events, 72 occurred in the subgroup with a prior TB history. In the group with a history of tuberculosis, COVID‐19 infection showed heterogeneity of effect and was associated with the risk of new tuberculosis treatment. Although only 0.5% of the total population had a history of tuberculosis, they accounted for about 6% of the incident cases. This is consistent with findings from a study in the Mexican border region, which reported that diabetes and COVID‐19 in the past 18 months synergistically associated with the risk of developing TB [26], as well as immunological reviews suggesting that temporary immunosuppression due to COVID‐19 or its treatment may promote reactivation of latent TB [27, 28]. This supports the possibility that COVID‐19 acts as a trigger that amplifies existing tuberculosis infections. On the contrary, individuals with glucocorticoid or DMARDs are well known to elevate infection risk, yet we did not observe an interaction with COVID‐19 beyond the baseline hazard (*p* = 0.21 for glucocorticoid, *p* = 0.98 for DMARDs). This may imply that although glucocorticoids can predispose to TB, the synergy effect with COVID‐19 is overshadowed by other factors, such as advanced age or direct post‐COVID immunopathology [29, 30].

Our results add a population‐based perspective, suggesting that the synergy is not restricted to severely ill, hospitalized patients but may apply across the broader community. Immune mechanisms likely underlie this association, especially involving alveolar macrophages and local inflammatory environments [31, 32]. The significantly higher hazard ratio among those with prior TB history points to alveolar or fibrotic lung changes that predispose to reactivation once the immune system is taxed by SARS‐CoV‐2 [27]. On the contrary, compared with previous reports indicating that a history of COVID‐19 alone did not significantly associate with TB risk [26], the significant fourfold increase in hazard observed in our overall results may reflect the large sample size and the heightened sensitivity from rigorous matching with healthy controls. The lack of significant interactions by sex or use of glucocorticoids/DMARDs contrasts with some regional studies which reported that TB reactivation was more common after severe COVID‐19 or steroid treatment but lacked clear evidence in milder cases [25]. This suggests that the combination of viral infection itself and prior TB lesions, rather than drug‐induced immunosuppression, may be the key risk factors.

Overall, this study provides large‐scale, nationwide quantitative evidence addressing the previously largely hypothetical issue [25, 28] of whether COVID‐19 has a biological impact on the development of TB. These findings should be interpreted in the context of national tuberculosis trends in Japan, where incidence has steadily declined and reached low‐incidence status (< 10 per 100,000) in recent years. Therefore, our findings do not suggest a reversal of national trends but rather indicate an elevated relative risk in specific high‐risk populations [7]. These findings highlight a subgroup that may warrant further clinical and epidemiological investigation.

### 4.1 Strengths and Limitations

This study has several important strengths. First, it utilizes a large, nationally representative sample of over six million matched individuals, enhancing the generalizability of the findings. Second, rigorous PSM was employed to balance key confounders, including age, sex, prior TB history, and use of glucocorticoids or DMARDs. Third, the primary outcome was defined as the co‐initiation of isoniazid and rifampin with new diagnosis code of tuberculosis related ICD‐10 codes, which was designed to minimize misclassification. This definition reduces the likelihood that TB prophylaxis would be mistaken for active TB treatment.

However, the study also has several limitations. First, there may be differential diagnostic intensity after COVID‐19 due to surveillance bias. Follow‐up visits and imaging procedures were more frequent in the COVID‐19 group than matched controls (Table S5). However, there are post‐exposure and intermediate factors between COVID‐19 and tuberculosis detection, and these were not adjusted in this study to avoid overadjustment. Second, as with all research based on administrative claims data, there is a risk of coding inaccuracies. Nonetheless, the requirement for concurrent prescription of multiple anti‐TB medications likely improves the specificity of TB case identification [33]. Third, we were unable to confirm TB cases microbiologically, as culture or PCR results were not available in the dataset. Fourth, residual confounding cannot be fully excluded; unmeasured factors, such as socioeconomic status, smoking history, geographic confounding, or severity of COVID‐19 illness may have influenced the observed associations. Fifth, the data were limited to the period ending in December 2022, so the effects of newer SARS‐CoV‐2 variants or longer‐term post‐COVID sequelae remain unknown. Lastly, the impact of non‐Japanese populations on TB risk in this cohort remains unclear. Although previous studies have suggested a transient decline in TB incidence among foreign residents during the pandemic, followed by a resurgence post‐pandemic, we were unable to explore this subgroup in detail within the current study framework [34].

Our findings suggest that COVID‐19 survivors, particularly those with any prior TB history, may represent a higher risk group that could warrant further clinical attention and investigation. TB control programs may warrant consideration in future studies and policy planning. Policymakers could develop integrated respiratory clinics where post‐COVID follow‐up includes symptom checks and targeted TB tests. As the pandemic evolves, ensuring robust TB surveillance in recovered COVID‐19 patients is vital to avoid surges in delayed or missed TB diagnoses.

## 5 Conclusion

In a nationwide propensity score–matched analysis, post‐COVID‐19 patients were associated with a fourfold higher risk of starting isoniazid‐based plus rifampin therapy. These data highlight a potential high‐risk group among COVID‐19 survivors, especially in older or populations with prior TB.

## Author Contributions

**Daisuke Miyamori:** conceptualization, investigation, writing – original draft, methodology, formal analysis, data curation, resources. **Sachi Nagasaka:** writing – review and editing, validation. **Masanori Ito:** validation, writing – review and editing, project administration, supervision. **Kotaro Ikeda:** validation, writing – review and editing.

## Funding

The authors have nothing to report.

## Disclosure

ICMJE statement: Contributors D.M. was responsible for the organization, coordination, and data analysis. K.I., S.N., and M.I. contributed to the writing of the final manuscript.

## Consent

The authors have nothing to report.

## Conflicts of Interest

The authors declare no conflicts of interest.

## Supporting information


**Table S1:** Covariates included in the propensity score matching.
**Table S2:** Definitions of Comorbidities and Treatments.
**Table S3:** Outcome definition with ICD‐10 codes for composite and each secondary outcome.
**Table S4:** Classification of Anti‐Tuberculosis Drugs.
**Table S5:** Frequency of follow‐up visits and imaging procedures among COVID‐19 and control group.

## Data Availability

The data that support the findings of this study are available on request from the corresponding author. The data are not publicly available due to privacy or ethical restrictions. The data for the study were obtained with ethical approval from Japan's Ministry of Health, Labor, and Welfare.
