# Supplementary material for: COVID‐19 as a Potential Trigger for Tuberculosis: Insights From a Large‐Scale Japanese Insurance Database Analysis
Source: J Gen Fam Med. 2026 Jun 2;27(4):e70139. doi: 10.1002/jgf2.70139 (PMC13240490; doi:10.1002/jgf2.70139)
Supplement: Supplementary file 1 — Table S1: Covariates included in the propensity score matching. Table S2: Definitions of Comorbidities and Treatments. Table S3: Outcome definition with ICD‐10 codes for composite and each secondary outcome. Table S4: Classification of Anti‐Tuberculosis Drugs. Table S5: Frequency of follow‐up visits and imaging procedures among COVID‐19 and control group. [file JGF2-27-e70139-s001.docx]

Supplementary Table 1. Covariates included in the propensity score matching

| **Category** | **Definition** |
| --- | --- |
| Age | 5-year category |
| Sex | 2 categories, male and female |
| CCI score | 4 categories, 0, 1, 2-3, and 4 or over. |
| Chronic pulmonary disease | Predefined ICD-10 codes describe in Supplementary Table 2 |
| Renal disease | Predefined ICD-10 codes describe in Supplementary Table 2 |
| Moderate or severe liver disease | Predefined ICD-10 codes describe in Supplementary Table 2 |
| AIDS/HIV | Predefined ICD-10 codes describe in Supplementary Table 2 |
| Diabetes | Predefined ICD-10 codes describe in Supplementary Table 2 |
| Malignancy | Predefined ICD-10 codes describe in Supplementary Table 2 |
| Health care utilization |  |
| Medical visit | Predefined ICD-10 codes describe in Supplementary Table 2 |
| Emergency visit | Predefined ICD-10 codes describe in Supplementary Table 2 |

Supplementary Table 2. Definitions of Comorbidities and Treatments

| **Category** | **Definition (ICD-10 or Drug Name)** |
| --- | --- |
| AMI | I21.x, I22.x, I25.2 |
| **CHF** | I09.9, I11.0, I13.0, I13.2, I25.5, I42.0, I42.5–I42.9, I43.x, I50.x, P29.0 |
| **PVD** | I70.x, I71.x, I73.1, I73.8, I73.9, I77.1, I79.0, I79.2, K55.1, K55.8, K55.9, Z95.8, Z95.9 |
| **CEVD** | G45.x, G46.x, H34.0, I60.x–I69.x |
| **Dementia** | F00.x–F03.x, F05.1, G30.x, G31.1 |
| **CPD** | I27.8, I27.9, J40.x–J47.x, J60.x–J67.x, J68.4, J70.1, J70.3 |
| **Rheumatic** | M05.x, M06.x, M31.5, M32.x–M34.x, M35.1, M35.3, M36.0 |
| PUD | K25.x–K28.x |
| **Diabetes** | E10.x–E14.x |
| HP/PAPL | G04.1, G11.4, G80.1–G80.2, G81.x, G82.x, G83.0–G83.4, G83.9 |
| **RD** | I12.0, I13.1, N03.2–N03.7, N05.2–N05.7, N18.x, N19.x, N25.0, Z49.0–Z49.2, Z94.0, Z99.2 |
| **Malignancy (excl. skin)** | C00.x–C26.x, C30.x–C34.x, C37.x–C41.x, C43.x, C45.x–C58.x, C60.x–C76.x, C77.x–C80.x, C81.x–C85.x, C88.x, C90.x–C97.x |
| **LD** | I85.0, I85.9, I86.4, I98.2, K70.4, K71.1, K72.1, K72.9, K76.5–K76.7 |
| **AIDS / HIV** | B20.x–B22.x, B24.x |
| **CCI score** | Calculated using status of 19 diseases, including AMI, CHF, PVD, CEVD, Dementia, CPD, Rheumatic, PUD, Diabetes, HP/PAPL, RD, Malignancy, LD and AIDS/HIV |
| **Prior TB history** | A15.x–A19.x |
| **Medications** |  |
| **Glucocorticoid user** | ≥4 prescriptions in past year for:  methylprednisolone, hydrocortisone, prednisolone, dexamethasone, betamethasone, mometasone. |
| **DMARD user** | ≥4 prescriptions in past year for:  thiomalate, penicillamine, bucillamine, iguratimod, salazosulfapyridine, actarit, leflunomide, tacrolimus, azathioprine, tofacitinib, upadacitinib, filgotinib, peficitinib, abatacept, etanercept, infliximab, adalimumab, certolizumab, golimumab, sarilumab, denosumab, tocilizumab, baricitinib |
| **Health Care Utilization** |  |
| **Medical visits** | Procedure codes for 111000110, 112007410 |
| **Emergency visits** | Procedure codes for 111000570, 111000670, 111000770 |

AMI, acute myocardial infarction; CHF, congestive heart failure; PVD, peripheral vascular disease; CEVD, cerebrovascular disease; COPD, chronic obstructive pulmonary disease; PUD, peptic ulcer disease; LD, liver disease; RD, renal disease; GC, glucocorticoid; DMARD, Disease-Modifying Antirheumatic Drug, TB; tuberculosis

If the ICD-10 codes registered before the index month, the participants were regarded as having the diseases. If the medication were used equal or more than 4 times per year before the index months, the participants were regarded as receiving medications.

Health utilization codes are defined as the total of each procedure codes during the 1-year pre-assessment period before the index month.

Supplementary Table 3: Outcome definition with ICD-10 codes for composite and each secondary outcomes

| **Outcome category** | **ICD-10 codes** | **Definition** |
| --- | --- | --- |
| Composite endpoint | A15.x–A19.x | Any tuberculosis diagnosis with treatment initiation |
| Respiratory tuberculosis, bacteriologically or histologically confirmed | A15.x | Respiratory tuberculosis confirmed bacteriologically or histologically |
| Respiratory tuberculosis, not confirmed bacteriologically or histologically | A16.x | Respiratory tuberculosis without bacteriological or histological confirmation |
| Tuberculosis of nervous system | A17.x | Tuberculosis involving the nervous system |
| Tuberculosis of other organs | A18.x | Extrapulmonary tuberculosis |
| Miliary tuberculosis | A19.x | Disseminated tuberculosis |

Tuberculosis treatment initiation was defined as concurrent initiation of rifampicin and isoniazid within the same billing cycle (or within 30 days), together with a tuberculosis-related ICD-10 code. The composite endpoint included any code within A15.x–A19.x, whereas secondary outcomes were defined using each ICD-10 category separately.

Supplementary table 4; Classification of Anti-Tuberculosis Drugs

| Drug Category | Generic Name |
| --- | --- |
| First-line drugs | Rifampicin, Isoniazid, Pyrazinamide, Ethambutol |
| Second-line drugs | Moxifloxacin, Amikacin, Kanamycin, Streptomycin, Enviomycin, Capreomycin, Cycloserine, Ethionamide, Delamanid, Bedaquiline |

Supplementary Table 5; Frequency of follow-up visits and imaging procedures among COVID-19 and control group

| Follow-up period after index date | Group | Visit | X-ray | Computed Tomography |
| --- | --- | --- | --- | --- |
| 0–2 months | Control | 57.6% | 6.8% | 2.3% |
|  | COVID-19 | 61.6% | 12.3% | 4.8% |
| 3–6 months | Control | 56.9% | 6.6% | 2.3% |
|  | COVID-19 | 66.4% | 11.0% | 4.5% |
| 7–12 months | Control | 57.2% | 6.5% | 2.3% |
|  | COVID-19 | 71.0% | 11.3% | 4.9% |
| 13–24 months | Control | 58.1% | 6.5% | 2.4% |
|  | COVID-19 | 78.2% | 12.1% | 5.5% |

Values indicate the proportion of participants with at least one event during each follow-up period.

Imaging procedures were identified using procedure codes from the Japanese national fee rules. X-ray procedures included codes 170001910, 170021750, and 170027910. CT procedures included codes 170011710, 170011810, 170028610, 170033410, 170034910, 170040210, 170040410, 170040610, 170040810, and 170041010. These codes represent plain radiography and computed tomography procedures regardless of detector configuration or facility type. Because the claims data do not provide information on the anatomical site of imaging, these procedures may include examinations unrelated to pulmonary evaluation.
